# Human milk sIgA antibody in relation to maternal nutrition and infant vulnerability in northern Kenya

**DOI:** 10.1093/emph/eoz030

**Published:** 2019-11-11

**Authors:** Masako Fujita, Katherine Wander, Nerli Paredes Ruvalcaba, Eleanor Brindle

**Affiliations:** 1 Department of Anthropology, Michigan State University, East Lansing, MI 48824, USA; 2 Biomarker Laboratory for Anthropological Research, Michigan State University, East Lansing, MI 48824, USA; 3 Department of Anthropology, Binghamton University (SUNY), Binghamton, NY, USA; 4 Laboratory for Anthropometry and Biomarkers, Binghamton University, Binghamton, NY, USA; 5 Center for Studies in Demography and Ecology, University of Washington, Seattle, WA, USA

**Keywords:** secretory immunoglobulin A, mid-upper arm circumference, vitamin A deficiency, iron-deficiency anemia, maternal buffering hypothesis

## Abstract

**Background:**

The maternal buffering hypothesis posits that human lactation biology can buffer milk against the mild-to-moderate malnutrition that occurred routinely in evolutionary history through the mobilization of maternal body reserves. This perspective may provide insights for understanding human milk immune content variation, such as milk sIgA, which protects infants’ intestines from microbial colonization and prevents diarrheal disease.

**Objective:**

To investigate how maternal delivery of sIgA to milk may vary in a way that can buffer milk against maternal malnutrition, while taking into consideration infants’ varying needs for immune protection across age or by sex.

**Methodology:**

A cross-sectional study analyzed archived milk specimens from breastfeeding mothers in Ariaal communities of northern Kenya surveyed during the 2006 Horn-of-Africa drought. Multiple regression models for ln-transformed sIgA were constructed using maternal nutrition, infant age/sex and their interactions as predictors. Maternal nutrition variables included iron-deficiency anemia (IDA), vitamin A deficiency (VAD) and mid-upper arm circumference (MUAC). Infant vulnerability was considered high in young age and/or male sex.

**Results and implications:**

Milk sIgA did not significantly differ by maternal IDA. Milk sIgA increased with infant age and maternal MUAC (*n* = 202). Significant interactions were observed between infant age and maternal VAD and between infant sex and maternal MUAC, such that milk sIgA content was low for younger infants particularly among VAD mothers, while among mothers with low MUAC, sIgA was lower for male infants. Results imply that mothers’ ability to deliver/buffer milk sIgA may be lowered when nutritional stress is combined with high infant vulnerability to infection.

**Lay Summary:**

Human milk sIgA antibody content was low for younger infants among vitamin A deficient mothers. Among mothers with small arm-circumference, milk sIgA was lower for sons. Double burden of raising young or male infants with high needs for immune protection and being malnourished, might lower maternal sIgA delivery to milk.

## INTRODUCTION

Human mothers’ milk provides nutrients, immune factors, and other bioactive compounds that nourish and protect their infants [1]. The nutrients in human milk, and more generally the milk of primates, are buffered against short-term maternal nutritional fluctuations [1–4] via a combined strategy of drawing on both dietary intake (‘income’) and body reserves (‘capital’) of nutrients for milk synthesis [5]. (This sets primates apart from some other mammals, such as rodents, that sustain lactation primarily through an ‘income’ strategy [5].)

Here, we build on this buffering hypothesis to (i) evaluate whether human milk *immune* content (secretory immunoglobulin A, sIgA, antibody) may be similarly buffered against maternal nutritional stress, and, (ii) integrate the complementary hypothesis that milk nutrient or immune content increases in proportion to infant need (e.g. nutritional need, or risk for infectious disease; this has been referred to as the ‘protective’ hypothesis [6, 7]). To accomplish this, we evaluate whether and how maternal and infant characteristics may interact to affect milk sIgA content.

### Milk immunity

Risk for diarrheal disease and death is substantially lower in breastfed than non-breastfed children [8]. Immune factors in breast milk provide important protections for infants against infection, primarily at the intestinal mucosal tissues, the major entry points for the microorganisms infecting human beings. Infants are vulnerable to infections due to immature immune systems; in particular, their secretory immune system is nearly non-existent during a variable period after birth [9]. For this reason, we focus on milk secretory immunity, specifically sIgA antibodies, which constitute the largest antibody system of the human body [9]. Higher milk sIgA concentration lowers infants’ risk for diarrheal disease [6] and is linked to the long-term development of competent microbiota and intestinal immunity [10].

### Maternal buffering

The combined strategy of income and capital investment allows milk nutrients to be buffered against decreases in dietary intake (which likely occurred routinely in primate evolutionary history [1–4, 7, 11, 12]), because the body reserves can continue to nourish milk [13]. Maternal body stores of fat and minerals can be transferred to offspring via milk, buffering infants against short-term fluctuations in maternal nutrition [1, 13] and poor health [7]. In the presence of more chronic and/or severe maternal nutritional stress, this buffering may occur incompletely or in complex ways, depending on the extent and type of malnutrition and overall maternal health [7, 12, 14]. We have recently suggested that maternal buffering of milk nutrient content may range from none to partial to complete, depending on the source of nutritional and health stresses to the mother [7]. Extending this model from milk nutrient content to milk immunity, it seems likely that the extent to which milk sIgA is buffered against maternal nutritional stress will depend on the type of malnutrition—maintaining sIgA delivery to milk may be easy in the face of some types of nutritional shortfall, but impossible in the face of others.

Consensus is currently lacking regarding the impact of maternal nutrition on milk sIgA concentration [15–17]. Some studies suggest a reduction in sIgA content with maternal malnutrition [17], while others report no effect [16]. These conflicting findings may be due, in part, to the confounding effects of unmeasured aspects of maternal nutrition such as micronutrient or protein status. Body mass index (BMI), the most commonly utilized indicator of maternal nutrition, does not fully capture the complexity of nutritional status [18].

Animal models [19–21] suggest that sIgA or sIgA-secreting plasma cells in mucosal tissues (e.g. intestines) are compromised by vitamin A deficiency (VAD), iron deficiency [22] and protein deficiency [23]. Because lactating mammary glands are a part of the integrated mucosal immune system [9], it is likely that these deficiencies similarly decrease milk sIgA delivery. However, these model organisms are imperfect for testing the buffering hypothesis as it relates to human mothers.

### Maternal protection

The protective framework utilized here views milk immune content to reflect maternal effort to protect the infant against infectious diseases [6, 13]. Under this framework, milk immune value reflects maternal immunological memory in the local disease ecology, maternal competence to deliver milk immunity and infant needs for protection. As a species, we raise offspring with higher and more prolonged postnatal mortality risk than any other species of the order Primates [24, 25]. Human milk synthesis is a product of deep evolutionary heritage that we share with other primates, and of more recent human adaptation to nourish and protect young whose needs are high and variable. Therefore, maternal delivery of milk content should vary with differing infant needs to facilitate maternal reproductive success [12, 26–28].

Milk immune content does indeed seem to vary according to children’s needs: the number of leukocytes [29] and the concentration of lactoferrin in milk both increase in response to infant infection, while milk sIgA may be proactively calibrated to protect infants against infectious disease [6]. With this reasoning, all else being equal, sIgA delivery to milk should be greatest for those infants at most risk for infectious disease; these include younger infants, with less developed immune systems, and male infants, with heightened vulnerability to infectious disease [30, 31].

Previous studies on the effect of infant age (or time postpartum) generally report a high concentration of sIgA in the colostrum or milk for neonates, which tends to decline across the subsequent days/weeks/months, although this decline may be balanced by compensatory changes in milk production [18, 27]. Some studies report a rebound in milk IgA (highly correlated with *s*IgA) with prolonged breastfeeding, suggesting that maternal investment of antibodies to milk may be readjusted upwards as mothers breastfeed longer term [32, 33]. Miller and McConnell [32] note that mothers in northern Kenya who had been breastfeeding for 18 months or longer produced milk with higher IgA concentration than those at 4–18 months. A longitudinal study among US women similarly reported a small rebound in milk IgA at 4 and 6 months postpartum compared with at 2 months postpartum [33].

### Maternal buffering and protection combined

**Figure 1.**
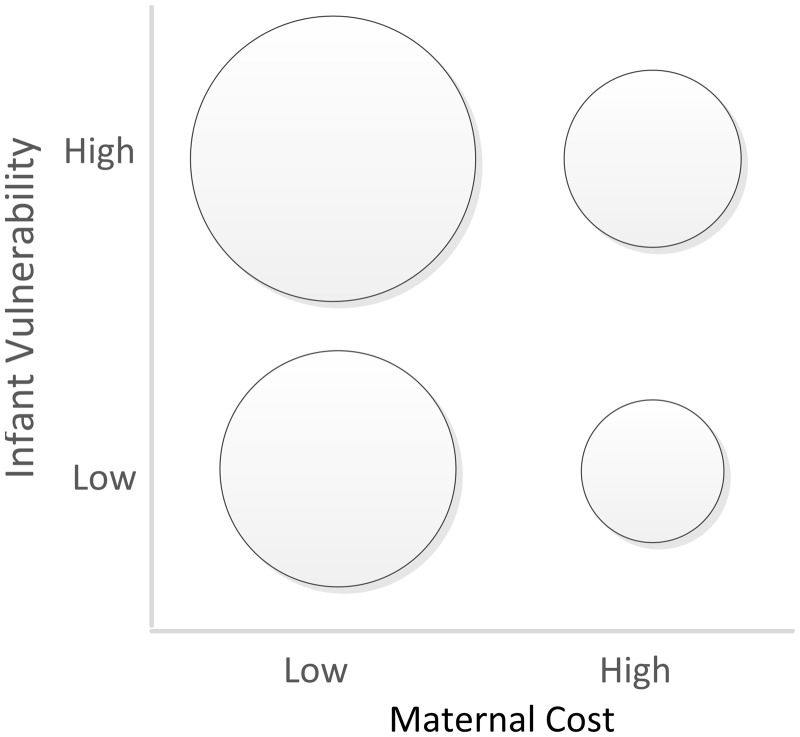
Hypothesized patterns in maternal sIgA delivery to milk by maternal cost and infant vulnerability to infectious disease. The framework utilized in this study predicts the highest milk sIgA delivery in the combination of low maternal cost and high infant vulnerability, and the lowest milk sIgA delivery in the combination of high maternal cost and low infant vulnerability. Maternal costs are high in the presence of malnutrition. Infant vulnerability is high for younger and/or male infants. sIgA, secretory immunoglobulin A

We expect milk sIgA to be buffered against maternal nutritional stress, and to reflect maternal protective effort. We generally predict that mothers raising infants with higher vulnerability (younger/male) deliver more sIgA to milk. We further expect that buffering sIgA against some nutritional deficiencies may come at high cost to mothers, or may be impossible; therefore we generally predict that mothers with nutritional deficiencies—protein-energy malnutrition (PEM), VAD and iron-deficiency anemia (IDA) [17, 19–23, 34]—imperfectly buffer milk sIgA delivery. Finally, we predict that the degree of sIgA buffering varies by infant vulnerability, with more robust buffering for infants who are more vulnerable to infectious disease. These predictions are summarized in Fig. 1. Specifically, we expect the following:

1. Nutritionally replete mothers with younger/male infants will produce milk higher in sIgA concentration than nutritionally replete mothers with older/female infants (due to higher protection for more vulnerable infants).
2. Mothers with VAD, PEM or IDA with younger/male infants will produce milk lower or equivalent in sIgA than nutritionally replete mothers with older/female infants (due to more robust but imperfect buffering against malnutrition for more vulnerable infants).
3. Malnourished mothers (VAD, PEM or IDA) with older/female infants will produce milk with the lowest sIgA (due to less robust buffering against malnutrition for less vulnerable infants).

## METHODOLOGY

### Study setting

A cross-sectional study of archived human milk specimens and associated data from marginally nourished mothers ≥18 years of age was conducted (*n* = 205). The specimens/data originated in Ariaal agropastoral communities of northern Kenya, where IDA, VAD and PEM are prevalent, and infectious morbidity and mortality are high [35–37]. The sociocultural, environmental, and ecological settings of these communities have been described elsewhere [38–41]. The data collection coincided with the 2006 Horn of Africa drought and associated food crisis [26, 42]. The mothers included in the present study were a convenience subsample (having complete data for relevant variables) of a random sample of 241 consenting mothers enrolled in the original research. The institutional review boards of the University of Washington and Kenya Medical Research Institute approved the original study. No further approvals were necessary for the present study of de-identified data and milk specimens.

### Data

#### Milk sIgA

The milk specimens were foremilk samples manually collected in the morning after an overnight fasting as described in depth elsewhere [26]. Upon collection, milk specimens were maintained frozen at cryogenic temperatures, except for thawing twice for the original research. Using thawed and homogenized archived milk specimens, sIgA concentrations were determined by an ELISA kit (Cat. no. RIC6100R, Biovendor, Ashville, NC, USA). sIgA was determined in the aqueous fraction of milk, obtained by removing cream and solids through triple-centrifuging at 4°C in the Biomarker Laboratory for Anthropological Research at Michigan State University in 2017. The intra assay CVs were <10% and the inter-assay CVs were ≤5% for the controls of low and high concentrations across seven plates.

#### Maternal protein-energy nutrition

The anthropometric indicators of the body’s protein-energy store available to this study included BMI, mid-upper arm circumference (MUAC), and triceps skinfold thickness (TSF). In this study of a marginally nourished population higher values of BMI, MUAC or TSF were considered to represent generally superior protein-energy nutritional status [43].

#### Maternal IDA and VAD

Data on IDA and VAD, the two leading public health nutritional problems in Kenya, particularly among mothers and children [44], were also available. The blood collection and laboratory methodologies we used to characterize IDA and VAD have been described elsewhere [45, 46]. In short, IDA was defined as Hb <12 g/dl in the presence of iron deficiency. Iron deficiency was identified using elevated transferrin receptor (>5 mg/l in dried blood spots) as the criterion. Low blood serum retinol (<30 µg/dl or <1.05 µmol/l) defined VAD (deficient = 1/not deficient = 0).

#### Infant age and sex

Infant age and sex variables were based on an interview with mothers. Infant age was calculated by counting the days from the infant’s date of birth to the date of interview. For descriptive purpose, infant age through the end of 4 months (<150 days) was defined as ‘young’ infants. Infant sex was reported by the mother, and coded as daughter = 0/son = 1.

#### Adjustment variables

Factors previously reported to have relationships with sIgA or maternal nutrition were utilized as adjustment variables. These included maternal age and parity, breastfeeding frequency, milk total protein (based on microBCA assay) and community. These variables were available from the original and more recent research [7, 42, 47]. Milk volume was not measured in the original research.

### Statistical analysis

Descriptive statistics were computed, and milk sIgA was natural log-transformed to remedy skewed distribution. The observed milk sIgA concentration values were compared by maternal nutritional status groups, both overall and stratified by infant age/sex groups. sIgA concentration values were then compared with reported values based on immunoassays from select populations.

To evaluate our predictions, a series of multiple regression models were constructed in three stages. In the first stage, models tested the effects of maternal nutrition indicators on sIgA. Maternal nutrition indicators included dichotomous variables IDA and VAD, and one of the continuous protein-energy nutrition variables (BMI, MUAC or TSF) in each model. Of these protein-energy nutrition variables, MUAC was most consistently associated with sIgA, and therefore subsequent stages of modeling focused on MUAC to investigate interactions with infant predictors.

In the second stage, infant age and sex were added to the model containing the three maternal nutrition variables (IDA, VAD and MUAC). In the third stage, models tested the interactive effects of maternal nutrition with infant age/sex, to allow for possible differences in maternal nutrient requirements or buffering for breastfeeding a son versus a daughter, or for breastfeeding infants of differing ages. Each interactive effect was tested while no other interactions were present in the model for the ease of interpretation. In all models, centered values of continuous predictors were utilized. All models were adjusted for covariates, initially including all the above-mentioned adjustment variables.

Post-regression, models were assessed for multicollinearity issues and undue influences of extreme values. Three sIgA outliers $(>\overset{-}{y}+3SD$) were identified to have undue influences, and were therefore removed from the models, making the final sample size *n* = 202. For models in which a significant interaction was observed, joint *F*-tests were performed to test the joint significance of the main and interaction terms.

Control for confounding was achieved by including all adjustment variables in regression models. Those that were not significantly associated with outcomes in any models were removed if Akaike’s information criteria [48] suggested they did not improve model fit. The α-level was set at 0.05. Stata version 15 was utilized for statistical calculations and graph construction. Microsoft Excel was utilized for the figure for cross-population comparison.

## RESULTS

### Sample characteristics

**Figure 2.**
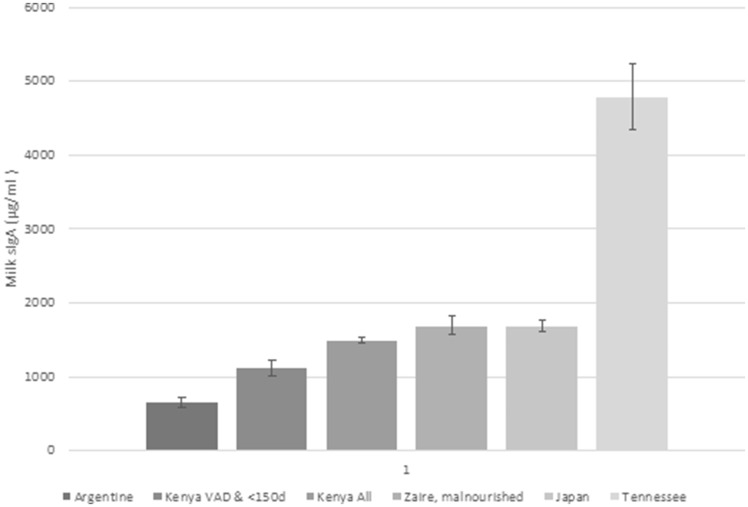
Concentrations of sIgA in mothers’ milk in northern Kenya along with published values from select populations. Kenyan values are shown in two bars: one for our sub-sample of VAD Ariaal mothers with young infants (<150 days), and the other for all of our participating mothers. The graph shows that, regardless of VA status, Ariaal mothers’ milk sIgA concentrations tended to occupy the lower range of reported values from several populations of differing levels/types of nutritional stress. Among the Ariaal, VAD mothers with young infants had significantly lower sIgA than the overall mean, but higher than the value reported from the Toba of northern Argentine. sIgA, secretory immunoglobulin A; VAD vitamin A deficiency

Table 1 summarizes descriptive statistics for the final 202 mothers. The concentration of milk sIgA observed in the present study was roughly comparable to previously reported by others using immunoassays [6, 10, 16, 49, 50], but placed toward the lower range among the several populations, for which sIgA concentration values were available (Fig. 2).

**Table 1.** Sample characteristics

	Mean or *n*	SD or %	Range
Breast milk			
Milk sIgA (µg/ml)	1491.88	554.86	438–3073
Milk total protein (g/dl)	0.98	0.16	0.65–1.52
Mother			
IDA (*n*, %)	36	18%	
VAD (*n*, %)	30	15%	
Hemoglobin (g/dl)	12.93	1.78	7.3–16.8
Serum retinol (µmol/l)	1.48	0.43	0.40–3.04
BMI (kg/M^2^)	19.72	2.88	14.4–33.49
MUAC (cm)	24.28	2.57	16.8–36.60
TSF (mm)	14.72	5.88	2.3–42.7
Parity	3.64	2.24	1–12
Age (year)	28	6.85	18–46
Infant			
Infant age (d)	244	135	24–585
Breastfeeding frequency	9.25	4.13	3–30

*n* = 202.

sIgA, secretory immunoglobulin A; IDA, iron deficiency anemia; VAD, vitamin A deficiency; BMI, body mass index; MUAC, mid-upper arm circumference; TSF, triceps skinfold thickness.

Mothers were predominantly normal-to-underweight, with the mean BMI of 19.7 (range: 14.4–33.5). IDA, VAD and MUAC were not significantly associated with each other. Infant age spanned from 24 to 585 days, with the mean of 244 days.

Ariaal mothers with VAD with young infants (<150 days) had low milk sIgA concentration (mean ± SD, range: 1117 ± 333, 438–1593 µg/m, *n* = 10) compared with the literature (with the exception of Toba mothers of Argentina, a population likely nutritionally stressed; 648 ± 379 µg/m, *n* = 30) [6]). The mean for the Ariaal mothers overall (1492 ± 555 µg/ml, *n* = 202) was more similar to, but lower than, published values from Zaire (1690 ± 600 µg/ml, *n* = 17 malnourished mothers) [16], Japan (1692 ± 690 µg/ml, *n* = 81) [51] or the USA (4973 ± 3174 µg/ml, *n* = 50 [49]).

### Hypothesis test results

#### Milk sIgA and maternal nutritional deficiency

In all the first-stage models, IDA and VAD were non-significant inverse predictors, and the protein energy nutrition indicators (BMI, MUAC or TSF) were non-significant positive predictors. MUAC had the largest coefficient (unstandardized, *B *= 0.016, standardized, *β* = 0.118, *P *= 0.058), followed by BMI and TSF (*B *= 0.12, *β* = 0.102, *P *= 0.101 and *B *= 0.002, *β* = 0.035, *P *= 0.578, respectively). Table 2 summarizes the initial model (Model 1) and the subsequent larger models using MUAC (Models 2–3b).

**Table 2.** Regression models for breast milk secretory immunoglobulin A (ln transformed, *n* = 202)^a^

	Model 1				Model 2				Model 3a				Model 3b			
Predictors	Coef.	SE	*P*	*β*	Coef.	SE	*P*	*β*	Coef.	SE	*P*	*β*	Coef.	SE	*P*	*β*
Maternal nutrition																
Maternal IDA	−0.06	0.06	0.30	−0.07	−0.05	0.06	0.37	−0.06	−0.05	0.06	0.35	−0.06	−0.05	0.06	0.36	−0.06
Maternal VAD	−0.07	0.06	0.23	−0.08	−0.07	0.06	0.24	−0.07	−0.08	0.06	0.20	−0.08	−0.1	0.06	0.08	−0.11
Maternal MUAC^b^	0.02	0.01	0.06	0.12	0.02	0.01	0.03	0.13	0.02	0.01	0.05	0.12	−0.01	0.01	0.49	−0.06
Infant characteristics																
Infant age^b^					0.00	0.00	0.05	0.13	0.00	0.00	0.33	0.07	0.00	0.00	0.03	0.14
Infant sex (male)					−0.05	0.04	0.26	−0.07	−0.05	0.04	0.21	−0.08	−0.05	0.04	0.28	−0.06
Mother–infant interaction																
VAD×infant age									0.00	0.00	0.02	0.16				
MUAC×infant sex													0.05	0.02	0.00	0.26
Adjustment variables																
Milk total protein	1.06	0.14	0.00	0.05	1.12	0.14	0.00	0.53	1.12	0.14	0.00	0.53	1.13	0.14	0.00	0.53
Community																
Karare	Ref				Ref				Ref				Ref			
Korr	−0.02	0.07	0.7	−0.02	−0.01	0.07	0.88	−0.01	−0.02	0.07	0.77	−0.02	0.01	0.06	0.93	0.01
Kituruni	0.15	0.06	0.01	0.17	0.15	0.06	0.01	0.18	0.14	0.06	0.01	0.16	0.15	0.06	0.01	0.17
Constant	6.21	0.14	0.00	–	6.16	0.14	0.00	–	6.17	0.14	0.00	–	6.16	0.14	0.00	–

*β*, standardized regression coefficient.

a For respective model, *R*^2^: 0.27, 0.29, 0.30 and 0.31. Adjusted R2: 0.24, 0.26, 0.27 and 0.28. Mean VIF: 1.09, 1.10, 1.13 and 1.11.

b Centered values used for MUAC and infant age.

IDA, iron deficiency anemia; VAD, vitamin A deficiency; MUAC, mid-upper arm circumference.

#### Milk sIgA and infant age/sex

In Model 2, infant age and sex variables were added to the baseline maternal nutrition model containing IDA, VAD and MUAC. Here, infant age was a significant positive predictor (*B *= 0.0003, *β* = 0.128, *P *= 0.049) while male infant sex was a nonsignificant inverse predictor (*B* = −0.048, *β* =−0.069, *P *= 0.260). The addition of these infant variables increased the magnitude of the MUAC effect and pushed its statistical significance beyond the α-level (*B *= 0.018, *β* = 0.131, *P *= 0.035). In this model, IDA and VAD remained inverse and non-significant predictors.

#### Milk sIgA and maternal nutritional deficiency and infant age/sex

The third-stage models testing the interactive effects of maternal nutrition and infant age/sex identified the presence of a significant interaction between VAD and infant age (*B *= 0.001, *β* = 0.158, *P *= 0.017; Model 3a in Table 2), and another between MUAC and infant sex (*B *= 0.048, *β* = 0.262, *P *= 0.004; Table 2, Model 3b). There were no other significant interactions between infant age or sex and maternal MUAC, IDA or VAD (results not shown).

**Figure 3.**
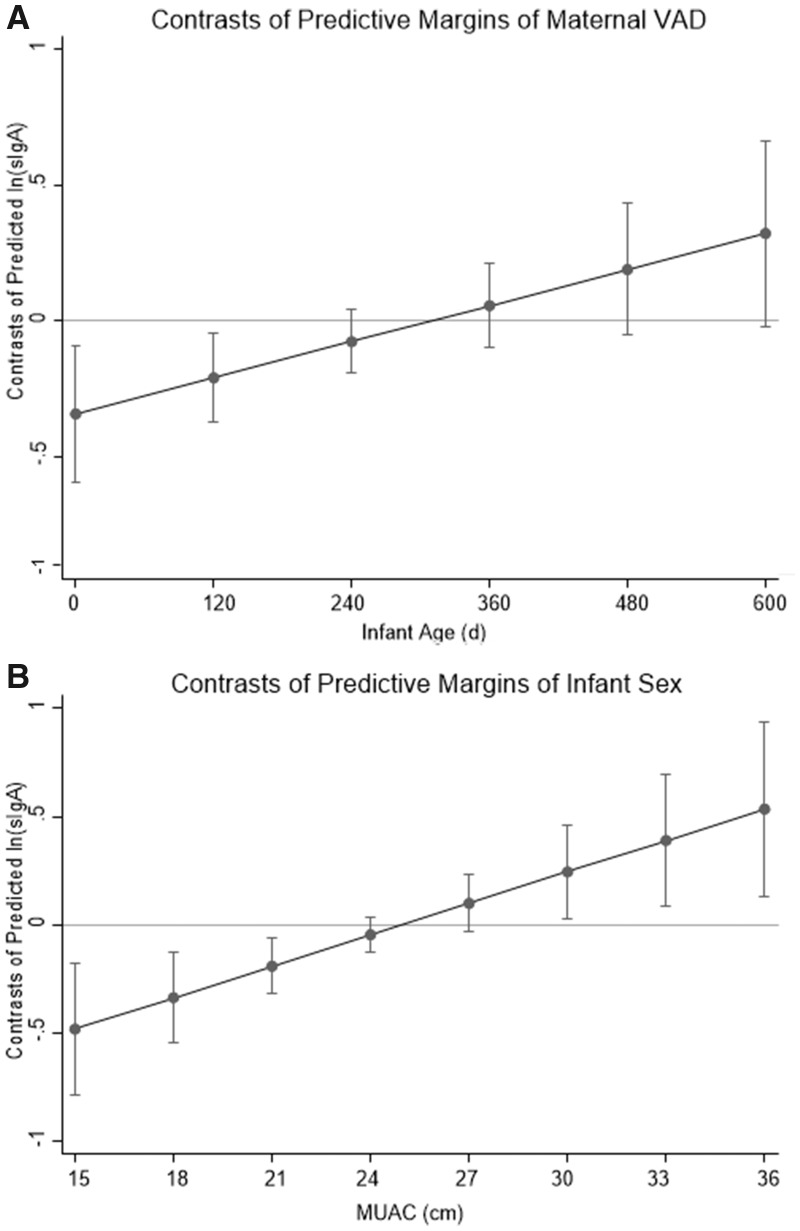
Contrasts of predicted sIgA (ln-transformed) between groups. (**A**) The difference between the predicted value for mothers without and with VAD (replete–deficient) across the infant age range. Milk from VAD mothers with younger infants (<150 days) was lower in sIgA concentration than VA replete mothers, but VAD mothers with older infants had milk sIgA comparable to VA replete mothers. (**B**) The difference between the predicted value for mothers with female and male infants (female–male) across the MUAC range. At lower MUAC, milk from mothers of male infants was lower in sIgA than milk from mothers of female infants; but, at higher MUAC, milk from mothers of male infants was higher in sIgA than milk from mothers of female infants. (The horizontal line at the value zero represents the equality of predictions between groups.) sIgA secretory immunoglobulin A; VAD vitamin A deficiency; MUAC mid-upper arm circumference

For the former interactive effect of VAD and infant age, the joint effect of VAD and the VAD-infant age interaction was significant (*F*_(2, 192)_ = 3.63, *P* = 0.028). The predictive margins plot for Model 3a (Fig. 3A) revealed the presence of a significant negative contrast toward the lower third of the infant age range (95% CI spanned below the value zero that represents the equality of predictions between groups, in this case between VAD and replete): milk sIgA was significantly lower among mothers with VAD than replete mothers if their infants were young, but sIgA did not differ significantly between the two groups if infants were older, whereas milk sIgA for replete mothers was relatively stable across the infant age spectrum. Taken together, these results indicated that VAD mothers of young infants had significantly lower concentrations of sIgA than either VA replete mothers of any infant age or VAD mothers of older infants, with or without adjustment for possible confounders (Table 3).

**Table 3.** Milk sIgA concentrations (µg/ml observed and adjusted) by maternal vitamin A status and infant age among Ariaal mothers in northern Kenya

sIgA mean (SD) range	Infant age	All mothers	VAD mothers	VA replete mothers
Observed values	All	1492 (555) 438–3703 (*n *= 202)	1367 (516) 438–2840 (*n *= 30)	1514 (560) 641–3703 (*n *= 172)
	<150 days^b^	1499 (417) 438–2847 (*n *= 59)	1117 (333) 438–1593 (*n *= 10)	1577 (391) 907–2847 (*n *= 49)
	≥150 days	1489 (604) 641–3703 (*n *= 143)	1492 (551) 795–2840 (*n *= 20)	1488 (614) 641–3703 (*n *= 123)
Adjusted values^a^	All	1430 (288) 797–2850 (*n *= 202)	1310 (288) 797–2159 (*n *= 30)	1451 (284) 988–2850 (*n *= 172)
	<150 days^b^	1456 (281) 797–2042 (*n *= 59)	1106 (159) 797–1425 (*n *= 10)	1527 (245) 1121–2042 (*n *= 49)
	≥150 days	1420 (292) 988–2850 (*n *= 143)	1412 (286) 988–2159 (*n *= 20)	1421 (294) 988–2850 (*n *= 123)

a Based on predicted values of Model 3a.

b Younger group defined as <30th percentile infant age (<150 days).

sIgA, secretory immunoglobulin A; VAD, vitamin A deficiency.

For the latter interaction between MUAC and infant sex the joint effect of infant sex and MUAC-infant sex interaction was significant (*F*_(2, 192)_ = 4.94, *P* = 0.008). The predictive margins plot (Fig. 3B) showed a negative contrast in the lower range (about MUAC <21), no contrast in the mid-range, and a positive contrast in the upper range (about MUAC ≥30). This indicated that mothers with low MUAC delivered less sIgA to milk for sons than daughters, but mothers with high MUAC delivered more sIgA for sons than daughters. In this model, the main effects of infant age turned greater in significance (*B *= 0.0004, *β* = 0.141; *P *= 0.027) compared with all other models. There was still no significant effect of IDA on sIgA. All of the above regression results were similar when three outliers with high influence were included.

## DISCUSSION

This study investigated how milk sIgA levels may vary in relation to specific aspects of maternal nutrition—namely IDA, VAD, and protein-energy nutrition—and infant age and sex using the frameworks of *buffering* infants against maternal malnutrition and *protecting* infants in proportion to their vulnerability to infectious disease. We predicted that sIgA in milk should be elevated among mothers raising young infants of either sex or raising sons, due to these infants’ elevated vulnerability. We further predicted that maternal buffering should generally protect milk sIgA against maternal nutritional stress; and, that as buffering becomes more costly, in the presence of IDA, VAD or PEM, it should be imperfect but more robust for more vulnerable infants. Results suggested that, overall, milk sIgA was buffered against the effects of maternal IDA and VAD, but that PEM might compromise milk sIgA delivery (Model 1). We did not observe extra protective effort in the form of elevated sIgA for young or male infants. Instead, milk sIgA was *positively* associated with infant age (Model 2). This was particularly true for mothers with VAD—those with younger infants delivered *less* sIgA to milk than mothers with VAD who had older infants—while vitamin A replete mothers delivered relatively constant levels of sIgA that were statistically equivalent to that of VAD mothers raising older infants (Model 3a). Finally, we found that infant sex had a significant interactive effect with MUAC: among mothers with sons, MUAC had a strong positive effect, such that milk sIgA increased significantly with MUAC. This was not the case for daughters. Thus, although these findings are consistent with our hypothesis of greater protection (elevated sIgA delivery) for male infants (among well-nourished mothers), it also suggests buffering in poorly nourished mothers (low MUAC) is greater for *daughters* than *sons* (Model 3b).

### PEM and male vulnerability

The sex-divergent MUAC-sIgA relationship may reflect sex-specific parental investment strategy [13, 52–54] and the environmental sensitivity of males [55]. Male prenatal growth tends to be more sensitive to insults; this may also be true during infancy and beyond [15, 31, 56]. Male infants are probably more susceptible to infections, and so we expected mothers to provide added immune protection for them, in the form of milk sIgA. Furthermore, due to this greater vulnerability, we expected mothers who are undergoing nutritional stress to buffer male infants more than female infants against compromised milk sIgA delivery. Thus, we expected to see the lowest milk sIgA among malnourished mothers of female infants, and the highest milk sIgA among well-nourished mothers of male infants. Instead, we found that mothers who had low MUAC (indicative of protein-energy nutritional stress) who were raising sons delivered the lowest sIgA, and mothers with higher MUAC who were raising sons delivered the highest sIgA. This does suggest some degree of greater protection against infectious diseases for sons, but may further suggest that buffering sIgA against maternal nutritional stress fails for sons to a greater degree than for daughters. This may reflect some higher nutritional cost to mothers of raising sons (e.g. higher energy cost for more energy dense milk [57]), or may be a strategy among malnourished mothers of investing more in children who are more likely to survive in harsh conditions (female infants, in this case [30, 31]).

### VAD and young infants

We expected to see protection and enhanced buffering of young infants, whose immature immune systems leave them particularly vulnerable to infectious disease. Instead, we saw lower sIgA among younger infants, particularly of VAD mothers, which could suggest imperfect buffering of younger infants in the presence of maternal VAD.

It is possible that we have mis-estimated the impact of age on infants’ risk for infectious disease in the environment of northern Kenya. If infants’ increasing independence and ability to move around the environment toward the older ages included in this study (approximately >1 year) results in *increasing* risk for infectious disease, the pattern we see of increasing sIgA with age might make sense for the protective hypothesis. This interpretation corroborates Miller and McConnell’s idea that milk IgA rebound may occur with prolonged breastfeeding because maternal investment in milk increases as infant age advances [10].

It is also possible that the observed patterns result from divergent maternal parental investment strategies [13, 52–54], with lower investment in vulnerable younger infants among VAD mothers compared with all other mothers as a strategy of decreasing investment in infants who are less likely to survive in the presence of VAD. Alternatively, lower milk sIgA among VAD mothers of young infants may reflect a high cost of breastfeeding younger infants, precluding buffering, perhaps because VAD is extra burdensome for mothers whose condition had not yet recovered from the physiologic load of pregnancy and childbirth [32, 58].

The interaction of infant age/sex and maternal nutrition in predicting sIgA levels does not necessarily mean that mothers *transferred* different amounts of sIgA antibodies to daughters vs. sons or infants of differing ages, because we did not take into account the influence of milk volume. Differences in milk volume by sex or age could have offset any differences in milk sIgA concentration. To the extent that breastfeeding frequency can serve as a proxy for milk volume transferred to an infant, however, this is unlikely; in our data breastfeeding frequency was equivalent for both sexes overall and across the infant age range for VAD group (the correlation between infant age and frequency was non-significantly positive; *r *= 0.025, *P *= 0.894, *n* = 31).

Similarly, lower sIgA delivery among VAD mothers raising young infants does not necessarily mean that their overall milk immune protection was compromised. Other immune factors, such as leukocytes or lactoferrin, which are usually abundant in milk early postpartum and can provide efficient protection for young infants [59], might have compensated for low sIgA and sustained immune protection for young infants of VAD mothers.

Nonetheless, given that maternal VAD is widely associated with elevated morbidity/mortality in children [60], the joint-effect of maternal VAD and the VAD-infant age interaction has implications for public health policies to combat infectious mortality among children of VAD mothers. In the existing literature, the main explanation for this association is that diminished transfer of *vitamin A* via milk, due to maternal deficiency [61], impairs infant endogenous immune function and development (both of which require vitamin A) [61]. Our findings suggest that the reduction in sIgA delivery to milk may also occur among VAD mothers in early postpartum months and this might create an additional pathway linking maternal VAD to elevated infant morbidity/mortality in some cases [62]. This calls for continued public health efforts to prevent VAD (and PEM) among mothers and women who may become mothers.

### Limitations

The present study suffers some limitations. First, infant age and sex were only two of the many proxies for the infant vulnerability. As such, this study was not able to explain some of the findings that may be due to unmeasured aspects of infant vulnerability or maternal condition. Second, this study did not collect information on milk volume consumed by infants. Third, we focused solely on milk sIgA, out of a host of milk immune factors. It is possible that other milk immune factors exhibit different patterns, and it is further possible that differences in patterns could be compensatory, such that the immune protection afforded by milk differs less across mothers than sIgA differs across mothers.

## CONCLUSIONS AND IMPLICATIONS

In the context of drought-related food scarcity of northern Kenya, we found that milk sIgA content was generally buffered against maternal VAD and IDA, but not against overall protein-energy nutritional stress (reflected in MUAC), and that some combinations of maternal malnutrition and infant vulnerability (VAD and young infant age; low MUAC and male sex) were associated with significantly lower milk sIgA. Possible explanations for the combined effects of infant vulnerability and maternal nutritional stress include higher costs to mothers of producing milk for more vulnerable infants, and a maternal strategy of lower investment in infants who are less likely to survive in harsh conditions. We do not think that the complex and nuanced buffering of milk nutrients that we previously observed in this sample [7] comports with a strategy of reduced investment in vulnerable children. Therefore, we favor the hypothesis that younger and male infants, who are more vulnerable to infectious disease mortality, are in some way more costly to mothers, and that this cost impacts the extent of maternal buffering of milk sIgA against nutritional shortfalls in macronutrients and vitamin A. Future research to evaluate the interaction between maternal nutritional stress and infant vulnerability across ecological contexts will be valuable in discriminating between these hypotheses. Future research should also be undertaken to investigate the impact of maternal VAD and milk sIgA levels on young infants’ infectious disease risk, which is likely to be relevant to VAD supplementation guidelines.
